# A pressure solution flow law for the seismogenic zone: Application to Cascadia

**DOI:** 10.1126/sciadv.adi7279

**Published:** 2024-01-24

**Authors:** Donald M. Fisher, Greg Hirth

**Affiliations:** ^1^ Department of Geosciences, The Pennsylvania State University, University Park, PA 16802, USA.; ^2^ Department of Earth, Environmental and Planetary Sciences, Brown University, Providence, RI 02912, USA.

## Abstract

We develop a linear viscous constitutive relationship for pressure solution constrained by models of deformed metasedimentary rocks and observations of exposed rocks from ancient subduction zones. We include pressure and temperature dependence on the solubility of silica in fluid by parameterizing a practical van’t Hoff relationship. This general flow law is well suited for making predictions about interseismic behavior of subduction zones. We apply the flow law to Cascadia, where thermal structure, geometry, relative plate velocity, and Global Positioning System velocity field are well constrained. Results are consistent with the temperature conditions at which resolvable ductile strain is recorded in subducted mudstones (at depths near the updip limit of the seismogenic zone) and with relative plate motion accommodated completely by viscous deformation (at depths near the downdip limit of the seismogenic zone). The flow law also predicts the observed forearc tapering of slip rate deficit with depth.

## Teaser

A flow law for pressure solution is quantified and used to predict interseismic coupling characteristics of subduction zones.

## INTRODUCTION

**Fig. 1.**
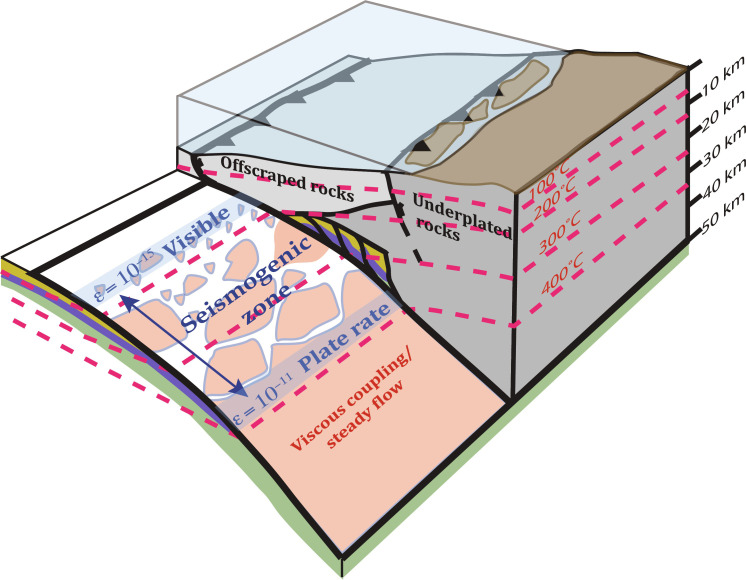
Block model of a subduction zone with a section of the forearc removed, exposing the top of the downgoing plate. Dashed red lines are isotherms. Pink patches represent locations of accelerated footwall deformation by diffusive mass transfer (DMT). Strain rate in footwall increases on average from the top to the bottom of the seismogenic zone, where steady strain occurs that accommodates the plate rate.

The subduction plate interface displays a wide range of slip behavior with increasing depth (Fig. 1) (*1*–*5*). The seismogenic zone, defined as the portion of the subduction interface where great earthquakes initiate, appears to coincide with a depth interval over which the slab-top temperature varies from 150° to 350°C (*6*–*8*). Previous work indicates that thermally activated ductile deformation also occurs at the conditions where these slip instabilities nucleate along the megathrust (*9*, *10*). The rheology and rate law associated with this deformation is relevant for earthquake dynamics because observations of interseismic slip deficits reflect a combination of plate motions and ductile, off-fault strain. This ductile deformation can lead to an evolution of the frictional behavior by affecting healing (i.e., an increase in cohesion) and/or permeability during interseismic intervals (*11*). Increasing rates of ductile deformation late in the seismic cycle could also result in resolvable precursor deformation. Here, we use field observations of ancient underthrust rocks to constrain a flow law for interseismic inelastic deformation. We apply the flow law using thermal models to compare predicted slip rate deficit with a geodetic inversion for the warm Cascadia subduction zone, which has a thickly sedimented incoming plate similar to that constrained by field examples from ancient subduction zones.

**Fig. 2.**
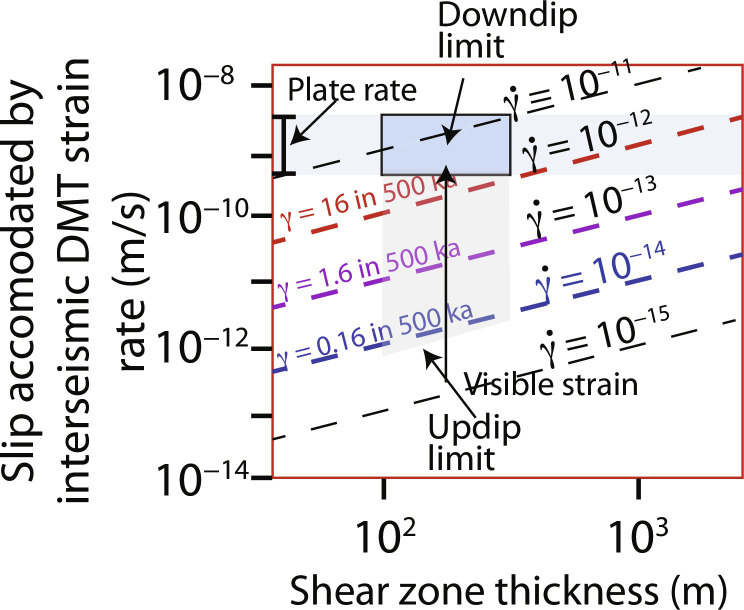
Graph of shear zone thickness (in meters) versus slip accommodated by DMT strain rate (in meters per second) contoured for strain rate. Blue band represents the range of plate rates, 1 to 10 km/Ma.

The relationship between relative plate motions and observed deformation fabrics can be directly related to subduction kinematics. In this framework, the properties of material above the interface are largely fixed, whereas the footwall of the interface undergoes downdip (spatial) and interseismic (temporal) changes in properties in response to inelastic deformation and increases in pressure and temperature during subduction. Given a typical slab-top geothermal gradient for a warm subduction zone such as Cascadia (*12*) and a displacement rate of 40 km/Ma, it takes 2 to 3 Ma to underthrust material through the seismogenic zone. Such a time interval could include thousands of earthquakes, each with more than a meter of slip. If an active shear zone in the footwall sediments was 100 m wide, then a steady ductile strain rate of ~10^−11^/s would be required to accommodate relative plate motions (Fig. 2). In contrast, a relatively fast geologic strain rate of 10^−13^/s in this shear zone is orders of magnitude too slow to completely accommodate plate motion (Fig. 2). Nonetheless, over many earthquake cycles, the ductile strain rate is high enough to affect the rock texture. For example, with a strain rate of 10^−13^/s, a large shear strain (γ) of ~1.6 would accumulate over 500 ka (i.e., the amount of time to underthrust a temperature interval of ~50°C). In this context, the slip rate deficit is defined by the difference between plate motion rate and the relative motion produced by ductile strain. The interseismic strain rate increases with increasing temperature between the updip and downdip limits of seismicity; in Fig. 2, the slip rate deficit is illustrated by the difference along the *y* axis between a given point and the blue box that represents the plate rates. An increase in interseismic ductile strain with depth leads to a tapering of the rate and amount of slip deficit accumulation along the plate interface. In the following section, we use inferences from rocks that were deformed along the subduction interface at conditions representative of the seismogenic zone to constrain the deformation mechanisms that control the form of the flow law that defines interseismic deformation.

### Record of underthrusting from exhumed paleosubduction interfaces in the Kodiak and Shimanto accretionary complexes

**Fig. 3.**
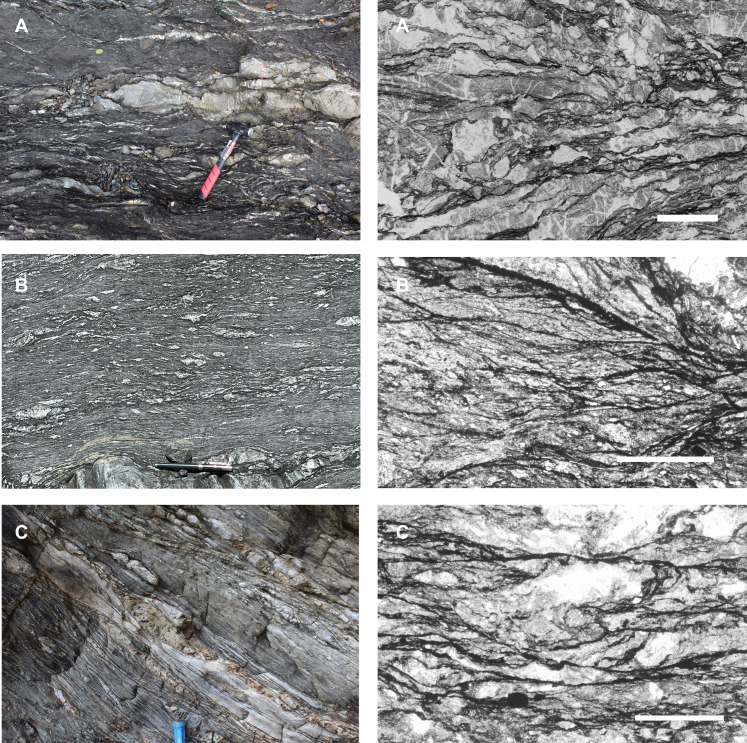
Field photos and photomicrographs of three melanges from the Shimanto Belt of Japan. (**A**) Lower Mugi mélange (~150°C) (*29*). Field photo (left) and photomicrograph (right, plane light) showing extensive veining in coarser grained lenses and anastomosing scaly fabric of dark insoluble residue. (**B**) Upper Mugi mélange (170° to 210°) (*29*). Scale bar, 500 μm. Field photo (left) and photomicrograph (right, plane light) showing anastomosing scaly fabric of dark insoluble residue, and (**C**) Makimine mélange (~350° maximum) (*30*). Field photo (left) and photomicrograph (right, plane light) showing anastomosing scaly fabric of dark insoluble residue. Scale bar, 500 μm.

The Kodiak accretionary complex and the Shimanto Belt of Japan expose rocks in the forearc of active convergent plate boundaries. Regionally extensive mélanges from these areas were transferred from the downgoing plate to the overriding plate at depths of seismogenesis during subduction of a sediment-rich plate boundary similar to the Cascadia and Nankai margins today (*9*). There are generally two different types of behavior recorded in rocks from all of these localities: (i) a wide (tens to hundreds of meters) zone of tectonic mélange that records diffusive mass transfer (DMT)–assisted noncoaxial strain (*13*, *14*); these rocks exhibit an anastomosing scaly fabric in mudstones and a pervasive network of veins in sandstone blocks (Fig. 3) (*15*–*17*); and (ii) a narrow (5 to 20 m) zone of cataclasis at the top of (*18*–*21*) or within the melange (*22*, *23*), which includes localized ultracataclasite and pseudotachylite (*22*–*24*). This dichotomy in style of deformation is consistent with slow interseismic ductile deformation (with strain rates below that necessary to accommodate plate motion) punctuated by earthquakes that lead to intense brittle deformation in a narrow zone.

Multiple lines of evidence support the interpretation that these mélange zone structures are representative of plate boundary deformation during underthrusting: (i) There is a ghost stratigraphy that is characteristic of the oceanic crust of subducting oceanic plates [e.g., (*16*)], with basaltic greenstones at the base overlain by either pelagic sediments or turbidites [the Uyak Complex (*25*)], Mugi mélange (*26*–*28*), Makimine mélange (*29*), and Yokonami mélange (*19*). (ii) Each of these examples is a regional shear zone; the Uganik Thrust, Uyak Complex, and Ghost Rocks mélange extend the entire length of the Kodiak Archipelago and hundreds of kilometers if correlative features on mainland Alaska are included. (iii) Kinematic indicators indicate that stratal disruption in the mélanges occurs during noncoaxial shear consistent with relative plate motions [e.g., (*16*, *17*)]. (iv) The shear zones are contained within thrust sheets that imbricate the oceanic crust during duplex accretion (*30*), with imbricate faults marked by basaltic greenstone in the hanging wall [for example, the Mugi (*17*, *29*, *31*), the Makimine (*29*), and the Uyak (*25*) mélanges]. Thus, the structures and fabrics of these shear zones predate accretion, and the complicating effects of exhumation-related deformation and retrograde metamorphism can be distinguished from the deformation that caused stratal disruption and scaly fabric development during underthrusting along the plate interface.

The field observations also provide details that can be used to characterize the grain-scale deformation processes. For example, noncoaxial deformation in the mélange is accommodated through slip on an anastomosing array of microshear zones that make up a scaly fabric (Fig. 3). The scaly fabric is defined by phyllosilicate-rich structures that show low concentrations of mobile elements and high concentrations of immobile elements as a result of dissolution along slip surfaces (*14*, *32*). The strength of lithologies in the mélange is heterogeneous; sandstone blocks are stronger than the mudstone matrix and deformed by coaxial opening mode cracking and sealing to form veins. Geochemical observations indicate local redistribution of mobile elements, with dissolution of quartz and calcite in shearing mudstones (leading to concentration of phyllosilicates that define the scaly fabric) and precipitation in cracked sandstones (*32*). These observations are all consistent with deformation by dissolution-precipitation creep, a mechanism that can be described by linear viscous flow and often is referred to by the catch-all term “pressure solution.” Microstructural observations indicate that the plate boundary shearing by this mechanism at relatively low temperature is restricted to fine-grained mudstones, while coarser-grained sandstone blocks only show evidence for lower strength and ductile deformation in the warmest mélanges (*29*). Deformation within these mélange units can be linked to the seismogenic *P*-*T* conditions of active subduction zones based on estimates of temperature. Constraints on temperature from these mélange units, primarily derived from analysis of fluid inclusions within veins and vitrinite reflectance, vary from 125° to 195°C in the Lower Mugi mélange (*33*) to a maximum of 300° to 350°C in the Makimine mélange (*34*). The thickness of the active shear zone in the footwall is difficult to constrain precisely, in part, because the shear zone is telescoped during duplex accretion after the development of the structural fabrics related to underthrusting. It also is not clear how much of the entire thickness of mélange was actively deforming at a given time. The mélange in thrust sheets of the Mugi mélange is less than 1 km in thickness. Structural reconstructions indicate that the active shear zone during underthrusting of subduction mélange was 100 to 350 m in thickness and maintained that thickness down dip within the seismogenic zone (*35*).

### Flow law for mudstones based on DMT in the temperature range of field examples

The importance of deformation involving pressure solution at lower-temperature conditions has been recognized for decades. While flow laws for pressure solution have been derived on the basis of theory [e.g., (*36*, *37*)] and analog experiments [e.g., (*36*–*39*)], experimental work on natural systems has led to a wide range of results. Furthermore, available flow laws for pressure solution predict a wide range of effective viscosity at conditions relevant to the subduction interface [e.g., (*10*, *40*, *41*)]. A complication for the experimental work is that the usual techniques used to enhance viscous deformation in the laboratory (imposing higher stresses and/or temperatures) are somewhat compromised by the low stress exponent and activation enthalpy for creep by pressure solution—both of which lead to the relative enhancement of dislocation creep (or brittle processes) at laboratory condition [e.g., (*36*)]. Furthermore, the range of temperature over which the natural systems can be studied is limited by the thermal stability of the phyllosilicate component of the natural systems. Last, some model parameters, such as grain boundary film thickness or island structure dimension, are not straightforward to estimate and may also vary with deformation conditions [e.g., (*37*, *42*)]. For these reasons, here, we leverage the previous work on natural rocks in which the rheology of phyllosilicate-rich siliciclastic sediments can be directly compared to the rheology of quartzite (*43*). In this case, the relatively well-constrained flow laws for dislocation creep of quartzite, which provide self-consistent results for laboratory and natural samples [within uncertainty, e.g., (*44*–*47*)] can be used to calibrate flow law parameters for pressure solution in rocks with synchronously deformed quartz veins and metasediments.

**Fig. 4.**
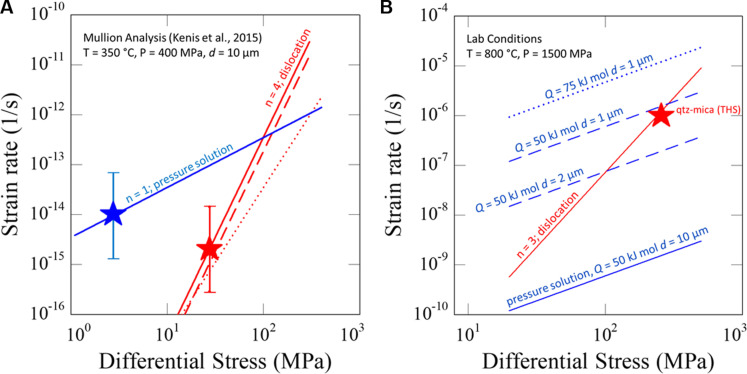
Graphs of differential stress versus strain rate under natural and laboratory conditions. (**A**) Pressure solution flow law based on the analysis of naturally deformed metasedimentary rocks. The red star shows conditions (and uncertainty) based on application of recrystallized grain size piezometry (*42*) and extrapolation of quartzite flow laws [solid line (*43*) and dashed and dotted lines (*46*)]. The blue star shows conditions for pressure solution from the analysis of (*42*). (**B**) Extrapolation of the pressure solution flow law back to laboratory conditions showing predictions for a range of activation energies (*Q*) and grain sizes. The red star shows the condition for a dislocation creep experiment on quartz + mica aggregate that exhibits microstructures indicative of pressure solution in regions of mixed recrystallized quartz and mica with a grain size of ~1 μm (*54*).

This technique was used to determine a flow law for pressure solution with the form $\overset{\cdot }{\epsilon }=A\prime \sigma^{n}$ by modeling a mullion structure with a well-constrained strain history and microstructures indicating pressure solution creep in the siliciclastic metasediments and dislocation creep in quartz veins (*42*). Kenis *et al.* (*42*) combined the well-documented strain distribution within the metasediments with a finite element deformation model to characterize the viscosity contrast between the quartz veins and the metasediments. A pressure solution flow law could then be derived with reference to the dislocation creep flow law for the quartz vein. The Kenis flow law, for which the *A*′ value and linear stress exponent (*n* = 1) were estimated for pressure solution, is shown by the blue star in Fig. 4A. For comparison, the flow laws used in the analysis of (*41*) predict much greater strain rates (on the order of 3^−10^/s) at the stress and temperature conditions illustrated for the blue star in Fig. 4A, whereas those estimated by (*10*) are in a similar range. The conditions for the red star for dislocation creep were determined using the recrystallized grain size for differential stress and a quartzite dislocation creep flow law from (*43*); essentially identical results would be determined using more recently calibrated flow laws [e.g., (*46*)]. To generalize this flow law for application to the subduction interface, we use a diffusion-limited flow law with the form

$$\overset{\cdot }{\epsilon }=\mathrm{AC}\sigma /d^{3}\text{exp}\left(-Q/\mathrm{RT}\right)$$ (1)

where *A*′ from (*42*) is substituted with *AC*/*d*^3^ exp(−*Q*/*RT*) to account for the temperature, grain size (*d*), and solubility (*C*) terms in the pressure solution flow law [e.g., (*36*, *37*)]. To calibrate the *A* term in this relationship, we use a grain size estimated from micrographs in (*42*) for the siliciclastic rocks, the temperature estimates for the deformation they studied, the solubility of quartz at their estimated pressure and temperature [from (*47*)], and a value of *Q* consistent with that determined for diffusion of silica in water at high pressure and temperature (*48*) and diffusion-limited dissolution of silica (*49*). The flow law parameters are *A* = 1.5 × 10^−6^ (for stress in megapascals, grain size in micrometers, and solubility in moles per kilogram) and *Q* = 50 kJ/mol; the relationship for *C* is outlined in the next paragraph.

**Fig. 5.**
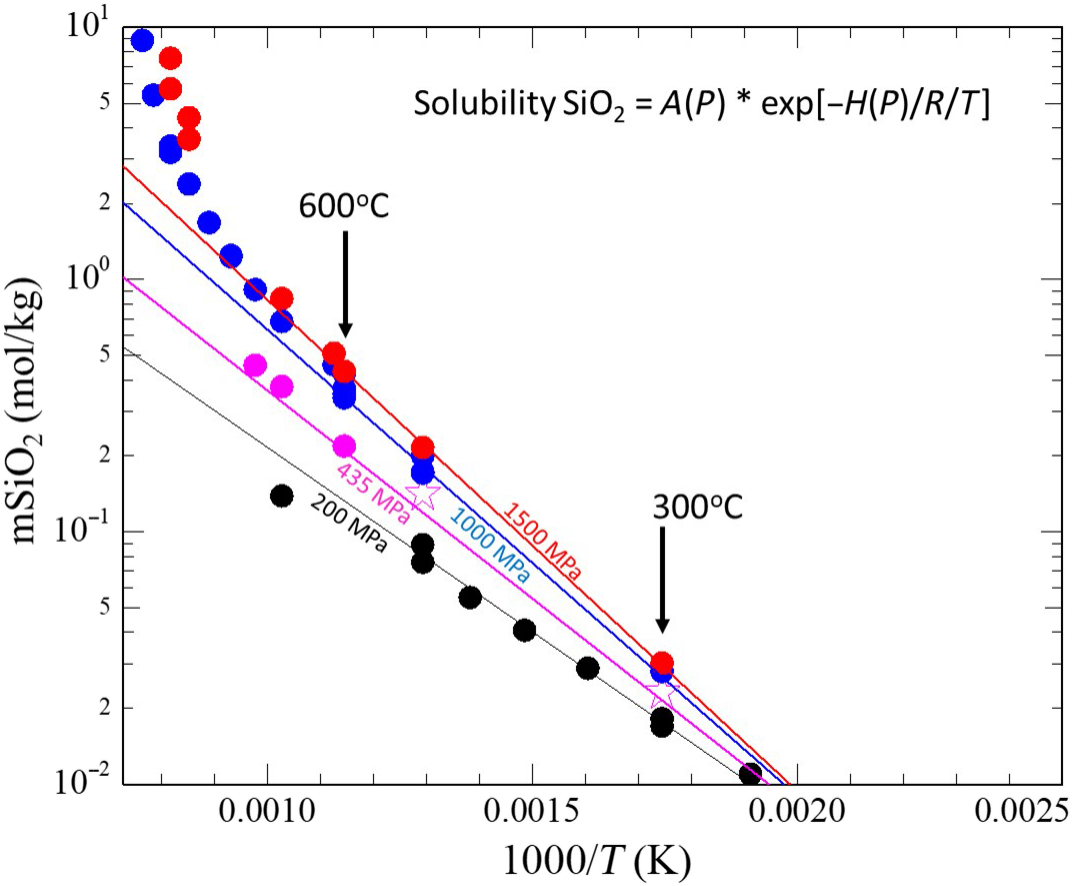
van’t Hoff plot showing the temperature dependence of solubility at different pressures. Data are fit to a relationship that includes a pressure-dependent activation enthalpy and preexponential factor. Data points are from (*45*), (*48*), and (*51*).

To apply the flow law to the range of conditions along the subduction interface, we also account for the pressure and temperature dependence of solubility (*47*, *50*, *51*). These effects are robustly quantified with relatively complicated empirical relationships, which, in turn, also include relatively complicated relationships for the equation of state of water. Therefore, we explored an empirical van’t Hoff relationship for the solubility of silica in water, *C*(*P*, *T*) [c.f., (*48*)] and found that data relevant to the conditions along the subduction interface can be well fit with a relatively simple relationship that simultaneously honors both the increase in temperature dependence observed with increasing pressure and the increase in pressure dependence observed at a higher temperature (Fig. 5): *C*(*P*, *T*) = *A*_sol_(*P*) exp[−*H*_sol_(*P*)/*RT*], for *C* in moles per kilogram and *H*_sol_ in joules per mole, where *H*_sol_(*P*) = 28,000 + 4500 ln(*P*/200 × 10^6^), with an uncertainty of ±1500 J/mol; and $A_{\text{sol}}\left(P\right)=0.0037\text{exp}\left[\frac{H_{\text{sol}}\left(P\right)}{R\times 453K}\right]$ ; note that *H*_sol_(*P*) = 28,000 at *P* = 200 MPa. The function provides an excellent fit to the data for temperatures up to ~700°C (i.e., below the critical point for the silica-water system at high pressure).

As a check on the resulting flow law, we compare the refined Kenis flow law for pressure solution with the dislocation creep flow law at high pressure and temperature laboratory conditions used to determine the dislocation creep flow laws (Fig. 4B). While pressure solution is not expected under these conditions for pure quartz aggregates (*52*), extrapolation of our field-constrained flow law to laboratory conditions indicates that pressure solution would be expected for very fine-grained aggregates of quartz and muscovite. The important role of intergranular phyllosilicate layers for promoting pressure solution has been demonstrated in analog experiments [e.g., (*38*, *41*, *53*)]. For comparison, microstructural observations on the recrystallized matrix produced during experiments on quartz + muscovite aggregates do show microstructural evidence for pressure solution (*54*); the conditions of those tests are highlighted in Fig. 4B.

**Fig. 6.**
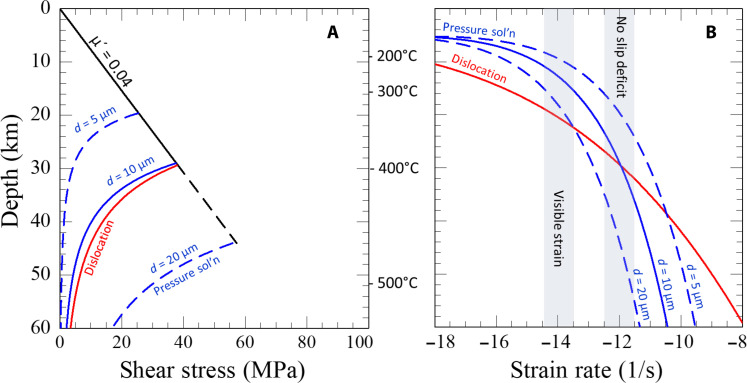
Shear stress and strain rate versus depth and temperature. (**A**) Shear stress in the brittle regime is calculated with an effective coefficient of friction of 0.04 (black line). Shear stress in the ductile regime is calculated for a strain rate of 3 × 10^−12^/s using the flow law for pressure solution at three different grain sizes (blue curves) and for dislocation creep (red curve) (*46*). (**B**) Strain rate versus depth and temperature. The light blue vertical bands show strain rates required to accommodate plate displacement within a 300-m-wide mélange zone (labeled no slip deficit) and to produce visible strain within a 300-m-wide mélange zone in a time interval at which *T* stays within a 50°C interval.

To explore the implications of the pressure solution flow law for understanding deformation processes in metasediments along the subduction interface, we calculate stress versus depth curves using slab-top temperatures determined for a thermal model of the Cascadia subduction zone (*55*). For the curves shown in Fig. 6A, predicted shear stresses are calculated using Eq. 1 for grain sizes of 5, 10, and 20 μm at a strain rate of 10^−12^/s (roughly consistent with the strain rate required to accommodate the relative plate motion in a 300-m-thick sedimentary layer). For comparison, the shear stresses using the lower-temperature quartz dislocation creep flow law from (*46*), which includes the pressure and temperature dependence of water fugacity [as parameterized by (*56*)], are also shown. The strength for the brittle regime is calculated using the relationship τ ≈ μ_*eff*_*P*_lith_, with an effective friction coefficient, μ_*eff*_ = 0.04 [equivalent to a friction coefficient of 0.6 with a near lithostatic fluid pressure (*P*_f_), i.e., λ = 0.95, where λ = *P*_f_ /*P*_lith_]. The low value for μ_*eff*_ = 0.04 along the subduction interface is constrained from analysis of heat flow and the moment tensors of upper plate earthquakes [e.g., (*57*, *58*)].

To apply the flow law to the subduction interface within the seismogenic zone, we calculate the strain rate as a function of depth using Eq. 1 and the shear stress consistent with μ_*eff*_ = 0.04 shown in Fig. 6A. As illustrated in Fig. 6B [c.f., figure 6 of (*41*)], substantial strain rates for pressure solution occur over the depth interval of ~10 to 30 km and depend strongly on grain size.

## RESULTS AND DISCUSSION

**Fig. 7.**
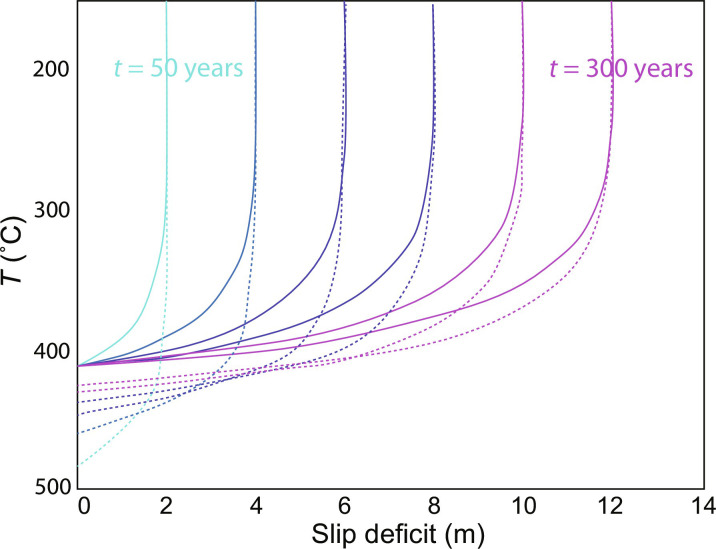
Subduction zone behavior over a seismic cycle based on the DMT interseismic flow law for accumulation of slip deficit. Time contoured in 50-year intervals from 50 to 300 years for two limiting cases: Differential stress remains close to the failure stress (solid curves) and the case where the differential stress increases to the failure stress over the course of the seismic cycle and all elastic strain is recovered after an earthquake.

The flow law predicts that pressure solution strain rate increases as temperature increases downdip along the plate interface. On the basis of the flow law, plate boundary deformation occurs at rates that produce a slip deficit but lead to measurable strains within the range of estimated shear zone thicknesses (i.e., 100 to 300 m) (*35*) at temperatures of around 200°C for fine-grained mudstones. For example, a γ of 0.16 would accumulate at a strain rate of ~10^−14^/s over the time interval in which the slab-top temperature changes from 175° to 225°C, which takes ~500 ka. This result is broadly consistent with the lower-temperature estimates obtained for melanges in the Shimanto Belt [i.e., the lower Mugi mélange (*33*)] and the observation that distributed pressure solution along scaly fabric is accompanied by brittle failure in sandstone blocks (*32*). With increasing depth (and increasing temperature), the pressure solution strain rate becomes high enough to accommodate the plate rate—the depth at which the slip deficit vanishes (Fig. 7). This location marks the downdip limit of the seismogenic zone, where weak lithologies accommodate deformation at the plate rate for a given thickness. The temperature at the downdip limit of slip deficit accumulation depends on the trade-off between plate rate and the shear zone thickness and the grain size. A wider shear zone can accommodate the plate rate at a lower strain rate (and thus lower temperature). The predictions for the temperature at the downdip extent of the seismogenic zone, calculated using the pressure solution flow law, is roughly consistent with observations from the warmest mélange (depending on grain size); the Makimine mélange (maximum of 300° to 350°C) (*34*) exhibits pervasive evidence for pressure solution in the mudstones and more ductile deformation of the sandstone blocks [although evidence for veins is still pervasive (*29*)].

Deformation at the downdip end of the seismogenic zone is complex, with low-frequency earthquakes, slow earthquakes, and afterslip that occurs in response to earthquakes that occur updip. Pressure solution is not capable of producing the fast strain rate transients (within bounds of expected slip rates and mélange thicknesses) related to slow earthquakes, which require another deformation mechanism (*10*). Nevertheless, the positive correlation between the flow law and microstructures preserved in the melanges that deformed in the seismogenic zone [and at conditions where slow slip is interpreted to occur (*10*)] provides a basis to quantify other parameters in the pressure solution flow law (e.g., by making comparisons of differences in strain analyzed at the outcrop scale), including grain size, the role of other phases such as plagioclase and calcite, and the role of synchronous deformation and reaction (i.e., incongruent pressure solution). Note that because the flow law is calibrated using the analysis of natural rocks, the relevant fluid had a “natural” composition, although the role of fluid composition could also be analyzed by making comparisons between rocks including a variation in the composition of fluid inclusions.

The flow law assumes that the diffusion length scale relevant for DMT within the scaly fabric microshear zones is the grain size, consistent with the observation that ductile strain is concentrated in the finer-grained mudstones. The grain size, determined through electron backscatter diffraction (EBSD), of metasedimentary rocks from the Makimine is 13 ± 7 μm (*59*); for comparison, these higher-temperature melanges deformed at a temperature similar to that of the rocks analyzed by Kenis *et al.* (*42*). Similarly, note that the strain rate calculated using the pressure solution flow law for a grain size of 10 μm is high enough to accommodate plate motion when the temperature increases to ~350° to 400°C (Fig. 6B). Sedimentary architecture and variation in the grain size of the fine-grained sediment fraction subducted along the plate interface could give rise to the heterogeneity in coupling inferred by both seismic inversions of moment release after large earthquakes (*60*, *61*) and geodetic inversions of interseismic coupling patterns (*62*–*64*). The flow law provides a mechanism for low coupling along margins with very-fine-grained sediment, with potential global impacts in the effect of grain size on interseismic coupling.

We reemphasize that our inclusion of the 1/*d*^3^ relationship in the flow law is primarily based on analog laboratory experiments and theory and is not yet determined experimentally at the conditions illustrated in Fig. 4B for the relevant lithologies. For comparison, in a range of experiments conducted on the compaction of salts, grain size exponents of ~3 are generally observed at conditions where the stress dependence is approximately linear [see table 5 of (*37*)], but there is considerable variation. If better constraints on grain size dependence become available, then it would be straightforward to include such effects by modifying the *A* value in the flow law to account for the difference in grain size exponent (using the value of 10 μm as a reference point).

The flow law also describes how strain rate along the interface due to pressure solution is affected by variations in stress. Figure 7 illustrates the effect of megathrust earthquake stress drop on the evolution of interseismic buildup of slip deficit with depth along a plate interface, for a plate velocity similar to Cascadia (i.e., 40 km/Ma). The dashed contours represent a case where the shear stress drops to 0 after the earthquake (total stress drop), whereas the solid contours represent the limiting case where the stress drop is negligible. After a coseismic stress drop, the pressure solution strain rate decreases abruptly, resulting in slip deficit accumulation to greater depths (and higher temperatures), but as the stress rises, the interseismic strain rate increases, and the downdip limit of slip deficit accumulation eventually returns to the point along the interface associated with steady deformation at the frictional strength. This downdip limit occurs at lower temperatures with slower plate motion, greater shear zone thickness, and decreasing grain size.

**Fig. 8.**
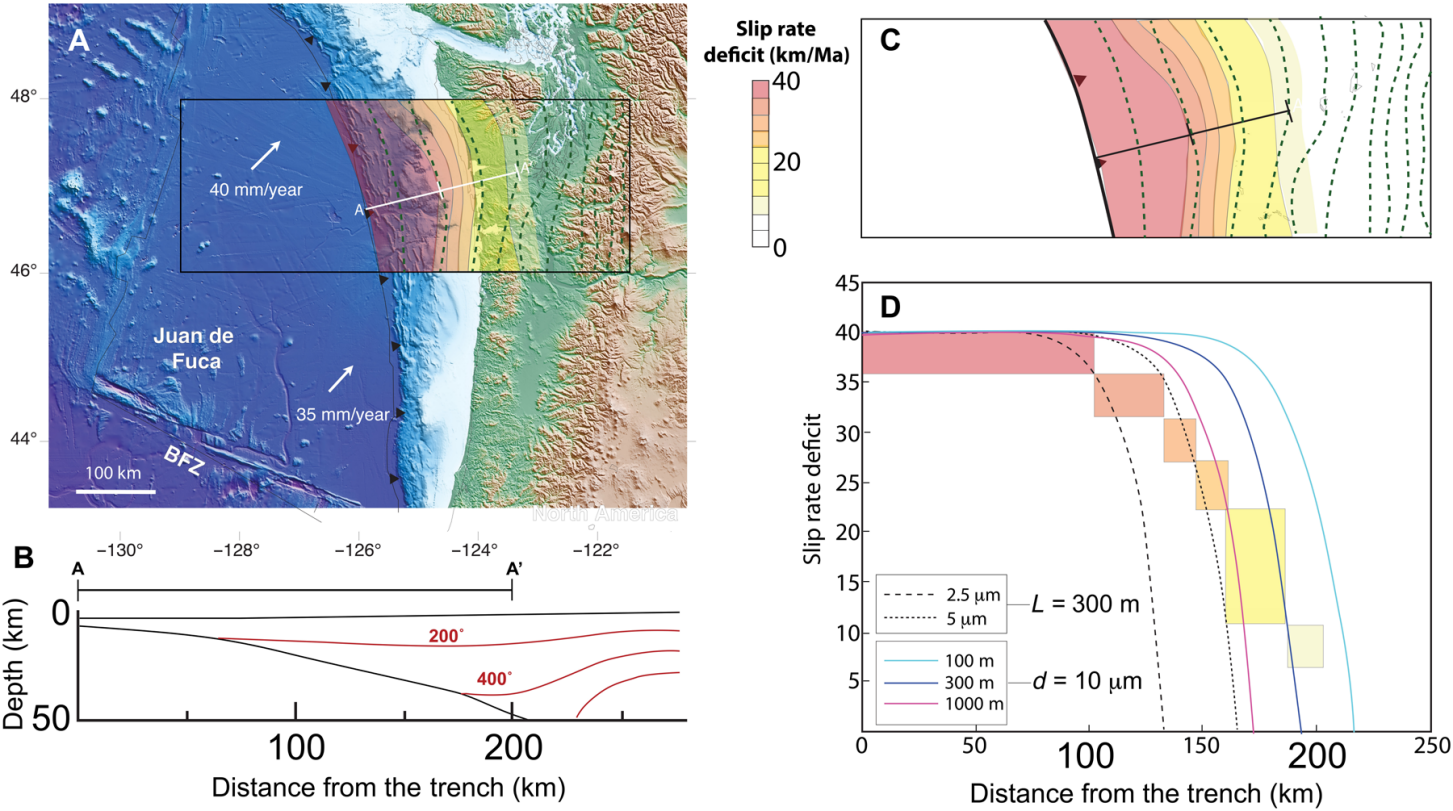
Application of the flow law to Cascadia. (**A**) Map of the Cascadia subduction zone showing topography, bathymetry, and location of the cross section. Dashed contours are the depth to the plate boundary (*74*). (**B**) Cross section showing 200° and 400° contours (*75*). (**C**) Top left: Variations in slip rate deficit based on inversion of GPS data (*63*). (**D**) Slip rate deficit, shown as a function of distance from the trench. Predictions of the flow law, with curves for different shear zone thicknesses (solid lines, a constant 10-μm grain size), and variable grain sizes (shown for a constant 300-m shear zone). Slip rate deficit of Cascadia from (C) is also shown (*73*, *74*).

Exposed examples of the subduction interface from the seismogenic zone are generally associated with elevated slab geotherms [e.g., (*65*–*67*)] and underthrusting of oceanic sediments (*17*, *68*). These characteristics are shared by Cascadia, a subduction zone that has a history of great earthquakes (*69*) and is hundreds of years into the interseismic period (Fig. 8A) (*70*, *71*). Cascadia is also an active margin with inversions of geodetic data (*72*, *73*) that can be compared with the predictions of the slip rate deficit calculated using the pressure solution flow law. Thermokinematic models for Cascadia (*57*), combined with estimates of the plate boundary geometry (*74*), predict that the 400°C isotherm resides at a depth of 30 km at a distance 170 km from the trench (Fig. 8B). On the basis of Global Positioning System (GPS) measurements, there is a tapering of slip rate deficit that begins ~100 km inboard of the trench and drops off to low values near the coastline, assuming viscoelastic behavior and 100% coupling near the trench (Fig. 8C) (*72*, *73*). The stress and temperature dependence of the pressure solution flow law predict a similar drop-off in the slip rate deficit with increasing downdip temperature (Fig. 8D). In this model, the amount of plate boundary slip that can be accommodated by interseismic pressure solution in the seismogenic zone is directly related to the shear zone thickness, with greater thicknesses accommodating the total plate motion at a lower temperature on the interface than narrower shear zones. Variations in grain size also modify the relationship between slip rate deficit and temperature due to the strong grain-size dependence of pressure solution; finer grain sizes lead to viscous accommodation of plate motions at lower temperatures and closer to the trench. In Cascadia, the downdip decrease in slip rate deficit is similar to the predictions of the simple model predicted with the flow law, with a departure from a fully coupled boundary about 110 to 120 km inboard of the trench. The landward misfit relative to the predictions of the model could be explained by coarsening of grain size near the downdip end of the seismogenic zone and/or narrowing of the shear zone in the footwall of the plate interface. This drift could also be explained by a slab top geotherm that is elevated relative to the predictions of the thermal model, for example, due to shear heating (*75*).

Pressure solution may also play an active role in the material properties that lead to slip instabilities. The seismic cycle requires restrengthening or healing after coseismic ruptures, with increasing healing rates with temperature in quartz-H_2_O systems (*76*). Healing is typically attributed to increases in either cohesion or coefficient of friction during holds (or interseismic periods) due to increases in contact area (*77*) that strengthen gouge linearly with log time (*78*). Dissolution-compaction (i.e., pressure solution) of a pervasive array of slip surfaces combined with sealing of crack spaces by precipitation is a potential macroscopic mechanism for rapid interseismic increases in contact area across the plate boundary zone.

Slip instabilities such as megathrust earthquakes occur when the plate boundary experiences slip weakening faster than the stored elastic strain can unload (*79*). Pressure solution is strongly velocity-strengthening (which would nominally inhibit the instability), so a different mechanism must lead to velocity weakening during nucleation of fast coseismic slip. It is noteworthy that velocity weakening of quartz-mica aggregates is observed during frictional sliding at slip rates of 1 μm/s at 150° to 350°C (*70*) (i.e., the relevant range for the seismogenic zone). The dichotomy between the tectonic mélange formed during underthrusting and the faults that cap the mélange likely preserves a record of these contrasting slip behaviors. Motivated by the similarity in predicted temperature at (i) the downdip limit of the seismogenic zone, (ii) the depth where the pressure solution flow law predicts a vanishingly small slip rate deficit for a reasonable sediment thickness, and (iii) the transition from velocity weakening to strengthening for quartz-mica aggregates (at a slip rate commensurate with that observed during slow slip events), we speculate that the rate of pressure solution creep also plays a role in processes that lead to the formation of localized frictional slip [c.f., (*10*, *80*)].

## MATERIALS AND METHODS

### Relationship between ductile strain and slip rate deficit

The assumptions in Fig. 2 are that ductile strain in the footwall of the plate interface is accommodated by simple shear parallel to the plate boundary. In this case, the shear strain rate is determined by the thickness of the shear zone and the velocity difference across the shear zone, expressed as slip rate accommodated by interseismic strain—a vector that can be subtracted from the relative plate motion vector to determine the slip rate deficit. The slip rate deficit reflects the coupling between lithospheric plates, which can be compared to the convergence rate of active plate boundaries. The strain rates shown on the plot depend on the slip accommodated by ductile strain and the width of the shear zone.

### Parameterization of the van’t Hoff relationship for solubility of silica

The solubility relationship illustrated in Fig. 3 was determined by fitting published data (*47*, *50*, *51*) for the solubility of silica in water as a function of pressure and temperature. The linear trends in the log(solubility) versus 1/*T* plots support the application of the van’t Hoff relationship (i.e., the temperature dependence of an equilibrium constant). The function we used to parameterize the change in the slope (i.e., activation enthalpy) as a function of pressure is empirical; the relationship fits the data much better than a relationship that includes an activation volume term and fits the published data well across the entire range of conditions we explore in our analysis.
